# Long-term trends in central obesity in England: an age-period-cohort approach

**DOI:** 10.1038/s41366-025-01949-5

**Published:** 2025-11-17

**Authors:** Laura A. Gray, Magdalena Opazo Breton

**Affiliations:** 1 Sheffield Centre for Health and Related Research, School of Medicine and Public Health, University of Sheffield, Sheffield, UK; 2 Healthy Lifespan Institute, University of Sheffield, Sheffield, UK; 3 Institution of Mental Health, University of Nottingham, Nottingham, UK

**Keywords:** Risk factors, Epidemiology, Ageing

## Abstract

**Background:**

Central obesity measures, such as waist circumference (WC), waist-to-hip ratio (WHR), and waist-to-height ratio (WHtR) have previously outperformed body mass index (BMI) in predicting health risks. BMI has been shown to underdiagnose obesity in older adults.

**Methods:**

We used data from the Health Survey for England (2005–2021) for 120,024 individuals aged 11–89 years, born in 1919–2008. High-risk classifications for WC, WHR, WHtR, and BMI were defined using established thresholds (World Health Organisation and the UK National Institute for Health and Care Excellence). Age, period (changes over time), and cohort effects were assessed using logistic regression with grouped variables to address the identification problem inherent in age-period-cohort (APC) models.

**Results:**

The prevalence of high-risk increased over time for all obesity measures. Central obesity measures showed a consistent linear increase with age until around 70 years of age. BMI exhibited an inverted U-shaped age trend. Obesity increased over time across all measures, while there was little evidence for a cohort effect. WHtR trends closely mirrored BMI at the population level but identified different high-risk individuals. The odds of high-risk WHtR increased with age, with odds ratios (OR) 4.91 (95% CI: 1.95–12.39) for females and 6.15 (95% CI: 2.24–16.89) for males by 85–89 years compared to 18–19 years. Period effects for WHtR showed ORs of 1.41 (95% CI: 1.16–1.72) for females and 1.25 (95% CI: 1.01–1.55) for males in 2019–2021 compared to 2005–2006.

**Conclusions:**

Central obesity measures, particularly WHtR, could provide a more consistent reflection of age-related increases in obesity risk. The linear increase in high-risk with age for central obesity measures aligns better with known age-related increases in obesity-related comorbidities. Age plays a significant role in driving obesity trends meaning an aging population could leading to further increases in the prevalence of obesity.

## Introduction

There is evidence that central obesity measures better identify health risks compared to more traditional measures of general obesity, such as body mass index (BMI) [1–3]. In particular, waist-to-height ratio (WHtR) [4], waist-to-hip ratio (WHR) [5] and waist circumference (WC) [6] have all been shown to outperform BMI in the early identification of health risks, in particular cardiovascular and cardiometabolic risks. There is also the concern that BMI can be problematic since it cannot distinguish between muscle mass and fat mass. This has been highlighted for children [7] and older aged groups [8–10]. Previous research has suggested that BMI could be misclassifying 10% of the UK population as having obesity when they do not, and that 25% of individuals with a healthy BMI may have been misclassified and therefore not alerted to their obesity-related health risks [11, 12]. It has also been shown that measuring BMI at a single point in time, rather than looking at patient history of BMI over their life course, could be inappropriate for identifying health risk, particularly in older adults [13].

The relationship between age and the prevalence of obesity, defined using BMI, follows an inverted U-shape, increasing throughout childhood and middle age but later declining into older age [14]. However, we know that the health risks associated with obesity do not decline as people age and so obesity defined using BMI does not reflect this continued increase in health risk. As well as this potential under-diagnosis in older adults, BMI has also been shown to significantly over-diagnose obesity in children^15^]. Additionally, the BMI threshold used to define obesity [16], as well as those used to define high-risk WC and WHR [17] differ by age and sex, making them more difficult to interpret and less transparent. In contrast, NICE suggest using the same WHtR thresholds for men, women and children [17, 18], making definitions easier to understand and aiding public health messaging. WHtR has also been shown to outperform other measures of obesity in predicting the risk of cardiovascular disease in adults [19, 20], trunk fat and total fat mass using dual energy x-ray absorptiometry (DEXA) measures in children [21] and liver steatosis and fibrosis in children and adults [22].

In response to concerns about the use of BMI alone in defining obesity, various proposals and guidelines and been developed recently. The National Institute of Health and Care Excellence (NICE) in the UK updated their obesity guidelines in 2025 [18], acknowledging the problems with using BMI alone and recommending the use of WHtR alongside BMI in individuals with a BMI less than 35 kg/m^2^. New definitions for obesity are emerging in an attempt to measure obesity in a way which better aligns with health risks and care needs. For example, new definitions of clinical and pre-clinical obesity have been developed by the Lancet Commission [10] and the European Association for the Study of Obesity (EASO) has outlined a new framework for the diagnosis, staging and management of obesity [23].

Long-term trends in general obesity and overweight, measured using BMI thresholds, have been investigated in England [14] as well as a variety of other settings [24–32]. However, there is less known about how the prevalence of central obesity measures change over time. Limited research into long-term trends in waist circumference has been conducted [33–35]. Similar research has been conducted for WHtR in Iranian children [36]. The literature on trends in central obesity measures lacks analysis over long periods of time or across wide age ranges, and often provides contradictory findings. More detailed research on how these measures change over time, and what drives this change, could provide insight into which measure(s) better reflect risk at an individual and population level.

Our study aims to disentangling the effects of age (life cycle), time (historical), and generation (cohort) over a 17-year period for adolescents and adults (aged 11–89 years) born between 1919 and 2008, to better understand the long-term trends in central obesity measures, and compare these trends to those of general obesity based on BMI following a previous study on the same population [14]. We follow an age-period-cohort (APC) approach previously used to study long-term trends in alcohol [37, 38], smoking [39] and general obesity [14]. Investigating the age effects for the prevalence of obesity using BMI and different central obesity measures, whilst accounting for period and birth cohort effects, will help determine which obesity measures are most appropriate for tackling obesity at different ages.

## Methods

### Data

We used data from the Health Survey for England (HSE) [40] between 2005 and 2021 (not including boost samples), which includes waist circumference measures for individuals aged 11 years and over. This is an annual cross-sectional survey collecting individual level data from English households with the purpose of monitoring population health. No data was available in 2020 due to the COVID-19 measures in place in England. The survey was conducted over the phone or video in 2021, while it was conducted in person all previous years [41]. We use data for cohorts born between 1919 and 2008 and individuals aged 11–89 years old. Data on central obesity measures in individuals below the age of 11 years were not available and were limited for individuals over 89 years.

### Outcome variables

We used three measures of central adiposity: waist circumference in centimetres (WC), waist-to-hip ratio (WHR) and waist-to-height ratio (WHtR). WHR was calculated using valid waist measurements divided by valid hip measurements. WHtR was calculated using valid waist measurements divided by valid height measurements. Waist circumference, hip and height were all measured in centimetres. BMI measures were calculated following Higgins and Marshall [42], dividing valid weight in kilograms by valid height in metres squared (kg/m^2^). Valid measures referred to entries where no problems were experienced obtaining reliable measures [41]. BMI, height and weight measures were self-reported for the first time in 2021. Adjustments were produced using prediction equations and self-reported information [41], but previous research suggests the adjusted version to be comparable to interviewer-measured [43]. In 2021, only a random selection of households had the nurse visit, which meant the number of observations for measures based on waist circumference was significantly lower than previous years [40].

Binary outcome variables were created in which individuals were classified into high (or substantially increased) risk categories using the definitions summarised in Table 1, giving the value ‘1’ to those individuals classified as ‘high-risk’, and the value ‘0’ otherwise. The World Health Organisation (WHO) states that there is a substantially increased risk to those who have a WC exceeding 102 cm in men and 88 cm in women [44]. For individuals younger than 18 years, thresholds calculated using the LMS method to align with adult thresholds are used [17]. The WHO also states that men with WHR 0.9 or higher and women with WHR 0.85 or higher are at substantially increased risk of metabolic complications [44]. The adult thresholds for WHR have been shown to be relatively stable throughout adolescence [17], and so these sex-specific thresholds were also applied to under 18-year-olds in this study. Current National Institute for Health and care Excellence (NICE) guidelines [18] state that a WHtR > 0.6 indicates high central adiposity. These thresholds apply to men, women and children [17]. These central obesity measures will be compared to general obesity using the well-established BMI threshold for obesity of 30 kg/m^2^.

**Table 1:** Adult high/substantially increased risk thresholds for central and general obesity measures.

	Adult thresholds	Childhood thresholds (11–17 years)
Waist circumference	>102 cm (men) >88 cm (women)	Determined by age and sex[17]
Waist-to-hip ratio	≥0.9 (men) ≥0.85 (women)	Determined by age and sex[17]
Waist-to-height ratio	>0.6	>0.6
Body mass index (BMI)	≥30 kg/m^2^	Determined by age and sex[16]

Using these risk-related thresholds, and their childhood and adolescent equivalent thresholds where appropriate for WC [17], WHR [17] and BMI [16], we created dummy variables, indicating high-risk in each of the measures, for use in the APC analysis. For each of the measures, we compared ‘high-risk’ to ‘non-high-risk’.

### Age-period-cohort variables

We predicted age in single years for data collected between 2015 and 2021 using a multiple imputation model. This was needed because only categorical age was provided in the HSE for these years, but single years of age were needed for the age and sex specific thresholds [16, 17], to create birth cohorts, and to study age trajectories. This method has been used in previous studies and more details on this method have been published elsewhere [14, 38].

To study age effects with our APC models, we used the categorical age variable provided in the HSE in our APC analysis with the group 18–19 years as the reference category. To study period effect, we grouped survey years into three-year periods, though the first group (2005–2006) and the last group (2019–2021) included only two survey years, since there was no survey in 2020 and 2004 could not be used because the sample of waist measures taken was considerably small. Finally, birth cohorts were computed using respondents’ age in years and survey years to create year of birth. Then, year of birth was grouped into five-year birth cohorts (1919–1923 to 2004–2008), a grouping common in APC analyses [45]. We observed 18 five-year birth cohorts. Individuals in the 2004–2008 cohort were only observed between 2015 and 2021, due to when they turned 11 years old.

### Identification in APC models

The linear relationship between age (A), period (P) and birth cohort (C) creates an ‘identification problem’ due to perfect multicollinearity between these variables [46, 47]. We used three strategies to deal with this issue. First, we used data visualisation for our outcome variables and the APC variables to understand patterns among long-term trends. Second, we group the APC variables in different sized groups to break the perfect multicollinearity, allowing the model to be identified. We assumed homogeneity within groups and heterogeneity between to find the effects of each APC variable. Finally, we used sensitivity analysis, modifying the selected groups to check the robustness of our results in relation to different grouping strategies.

### Statistical analysis

First, we plotted our outcome variables against survey years to graphically display the prevalence of high-risk general and central obesity over time. Next, we plotted age trajectories for each five-year birth cohort to show the prevalence of high-risk general and central obesity by birth cohort group as they aged. Lastly, we estimated four APC models, one for each of our binary outcome measure using our grouped APC variables. We estimated separate models for males and females to compare differences in trends by sex. Our APC models used logistic regression and were run using sample weights. We displayed our results graphically in odds ratios (ORs) and 95% confidence intervals (95% CI). The reference category for each APC variable was: 18–19 years for age, 2004–2006 for period, and 1919–1923 for birth cohort.

To ensure that our results were robust, we performed a sensitivity analysis using different periods and cohort grouping variables. We used ten-year birth cohorts and four-year survey periods, while keeping the same HSE age groups because the exact year of age was not provided in the HSE from 2015 onwards.

## Results

Our analysis, using the HSE, covered a study period of 16 years (2005–2021) of annual data, comprising 120,024 adults and children in England. No data was collected in 2020. Our sample contains data on individuals aged 11–89 across 17 five-year birth cohorts. The number of valid observations in each adiposity measure varied by measure. We had 84,033 valid WHtR observations, 88,331 valid WHR observations, 88,575 valid WC observations and 104,548 valid BMI observations. A flowchart illustrating the sample size for each measure can be found in the supplementary materials (Supplementary Fig. S1).

**Fig. 1:**
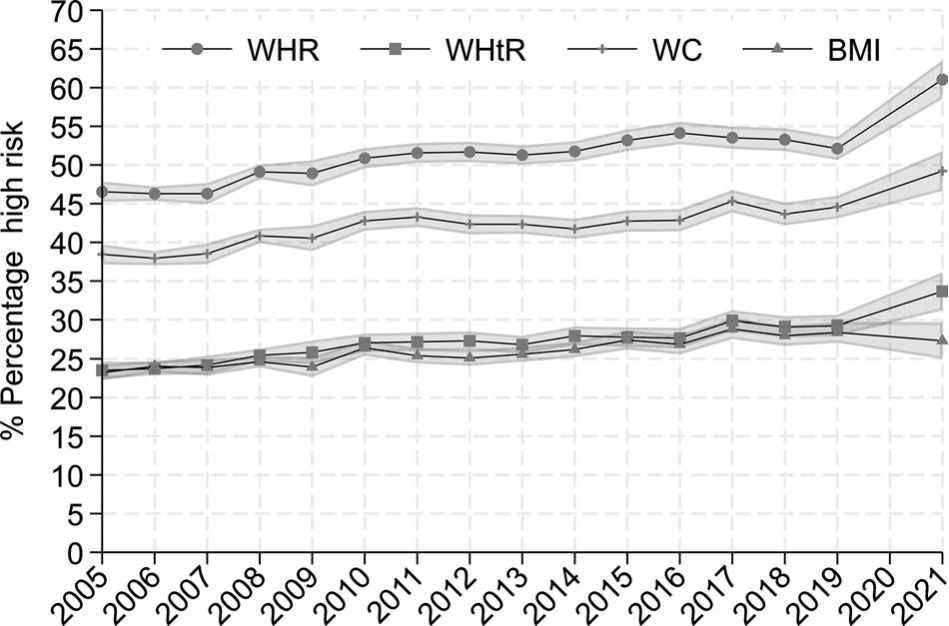
Trends in obesity using different measures. Percentage of the population at high-risk for waist-to-height ratio (WHtR), waist-to-hip ratio (WHR), waist circumference (WC) and body mass index (BMI); Health Survey England (2005–2021).

Figure 1 shows there was a steady increase in the prevalence of high-risk general and central obesity across all measures during our study period. The prevalence of high-risk BMI shows a steadily increasing trend, starting at 23.22% in 2005 (95% CI: 22.21–24.23%) and rising to 27.34% in 2021 (95% CI: 25.00–29.69%). High-risk WHtR showed a similar pattern, with only a slightly higher prevalence than high-risk BMI during the same period. The prevalence of high-risk WHtR was 23.52% in 2005 (95% CI: 22.37–24.68%), and raised to 33.38% in 2021 (95% CI: 30.97–35.79%). High-risk WC increased at a similar rate but showed a higher overall prevalence, rising from 38.45% in 2005 (95% CI: 37.17–39.73%) to 49.22% in 2021 (95% CI: 46.66–51.77%). High-risk WHR also showed an increasing trend but had the highest overall prevalence of high-risk, rising from 46.29% in 2005 (95% CI: 45.23–47.86%) to 61.04% in 2021 (95% CI: 58.54–63.53%). The prevalence for all measures based on waist measurement was significantly higher in 2021 than in all previous measures.

**Fig. 2:**
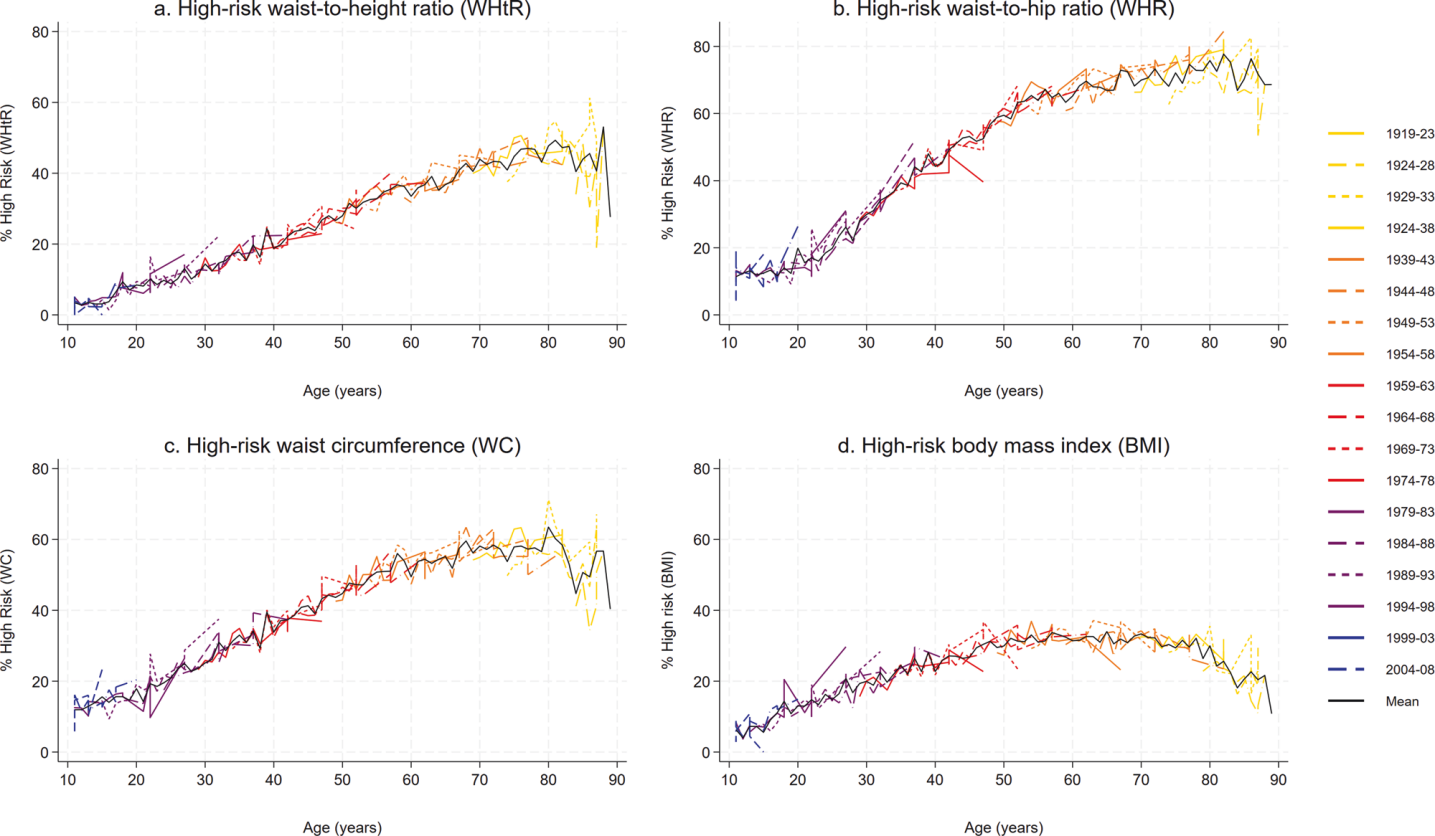
Age trajectories by five-year birth cohort. Age trajectories by five-year birth cohort for high-risk: **a** waist-to-height ratio (WHtR), **b** waist-to-hip ratio (WHR), **c** waist circumference (WC) and **d** body mass index (BMI), Health Survey England (2001–2021). Note: We used the same thresholds for high-risk WHtR for adults and children. For other measures we used specific sex and age thresholds in children.

Figure 2 shows the age trajectories for the prevalence of high-risk using general and central obesity measures. Each coloured line represents a five-year birth cohort. The prevalence of high-risk increased with age across all central general obesity measures. The central adiposity measures also showed a generally linear increase with age, which was more evident for WHtR and WC, but high-risk BMI showed an inverted-U shape with age. The prevalence of high-risk BMI increased in adolescents and into adulthood, but this increase slowed during middle age and plateaus at around the age of 50 years and even decreased in older adults over 65 years. The increasing age trajectory observed in high-risk WC and WHR appeared to level off at around 65–70 years. WHtR showed a clear linear increase from ages 11 to 85 and became more volatile at older ages, similar to the other measures, and likely due to lower sample sizes in older age groups. WHR was relatively flat from ages 11 to around 20 years old, then consistently increased until around 65 years, when the rate of increase slowed compared to earlier ages.

**Fig. 3:**
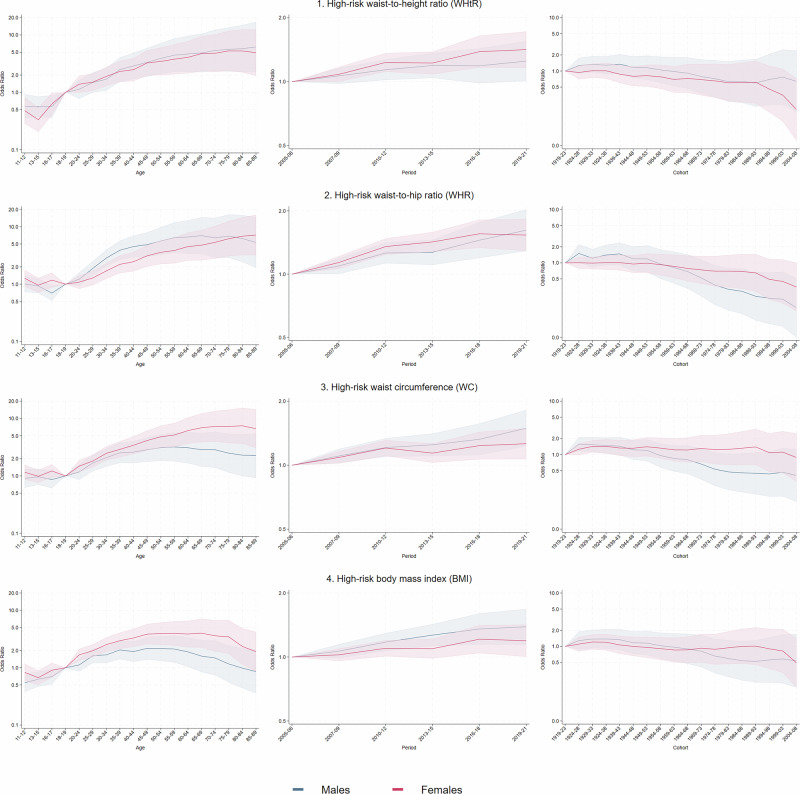
Age, period and cohort effects. Age, period and cohort effects, estimated odds ratio (OR) and 95% confidence intervals on displayed alogarithmic scale for high-risk in **1)** waist-to-height ratio (WHtR), **2)** waist-to-hip ratio (WHR), **3)** waist circumference (WC) and **4)** body mass index (BMI) by HSE age group, three-year survey period, and five-year birth cohort for males and females (Health Survey England, 2005–2021).

Figure 3 shows the age period and cohort effects for males and females, across each of the obesity measures, using our APC analyses. Full regression results can be found in the supplementary material: Supplementary Tables S1–S8 show the full APC regression results for WHtR, WHR, WC and BMI for both females and males, respectively.

### Age effects

The odds of having high-risk WHtR were lowest in the age group 11–12 (OR females: 0.48; 95% CI: 0.28–0.81; OR males: 0.57; 95% CI: 0.35–0.93), compared to the age group 18–19, and increased at similar rate until the age group 85–89 for females (OR: 4.91; 95% CI: 1.95–12.39) and males (OR: 6.15; 95% CI: 1.95–12.39), with no significant difference between males and females. The odds of having high-risk WHR were close to 1 during childhood, compared to 18–19 years, and similar for males and females. Then the odds increased almost linearly for females until age group 85–89 (OR: 7.12; 95% CI: 3.19–16.18), while for men they increased until age group 80–84 (OR: 6.24; 95% CI: 2.45–15.92) and decreased for group 85–89 (OR: 5.33; 95% CI: 1.93–14.70), compared to our reference category. Similarly, for the high-risk WC measure, the odds were also close to 1 in younger age groups compared to 18–19 years for both males and females, with no statistically significant difference. Then, the odds increased for males and females until 55–59 years in males (OR: 3.19; 95% CI: 1.80–5.67) and 80–84 years in females (OR: 7.48; 95% CI: 3.65–15.31), compared to our reference category, and slowly decreased afterwards. Finally, the odds of having high-risk BMI were lowest among males aged 11–12 (OR: 0.55; 95% CI: 0.38–0.79), while for females, the odds were lowest in the 13–15 age group (OR: 0.67; 95% CI: 0.50–0.89), compared to the age group 18–19. The odds then increased until the age group 50–54 in males (OR: 0.17; 95% CI: 0.32–3.56), and the age group 65–69 in females (OR: 4.00; 95% CI: 0.25–7.12), and decreased afterwards.

### Period effects

The odds of having high-risk WHtR increases slightly throughout the study period, with the largest odds observed in group 2019–2021 (OR females: 1.41; 95% CI: 1.16–1.72; OR males: 1.24; 95% CI: 1.01–1.55) compared to the reference category (2005–2006). A similar increase was observed for high-risk WHR, with the largest odds observed in group 2019–2021 (OR females: 1.53; 95% CI: 1.29–1.83; OR males: 1.62; 95% CI: 1.29–2.03), for high-risk WC (OR females: 1.26; 95% CI: 1.07–1.49; OR males: 1.49; 95% CI: 1.23–1.82), and high-risk BMI (OR females: 1.19; 95% CI: 1.00–1.42; OR males: 1.39; 95% CI: 1.14–0.69). No statistically significant differences were observed between males and females.

### Cohort effects

The odds of having high-risk WHtR were similar across birth cohorts, with only females in birth cohort 2004–2008 displaying odds significantly lower than 1 (OR: 0.19; 95% CI: 0.05–0.73). Interestingly, for high-risk WHR, it is only males in birth cohorts born after 1974 displayed odds significantly lower than 1 (OR 1974–1978: 0.38; 95% CI: 0.16–0.90). The odds of having high-risk WC were again similar across birth cohorts, with males and females displaying odds significantly higher than 1 for cohorts born between 1924 and 1938. No significant differences were observed in high-risk BMI across any cohorts investigated in this study.

### Sensitivity analysis

Results from our sensitivity analysis can be found in Supplementary Fig. S2 in the supplementary material. They showed very similar patterns across age, period and cohort effects, though differences across birth cohorts were smaller than in our main analysis.

## Discussion

This study used APC analyses to investigate long-term trends of ‘high-risk’ obesity using general and central obesity measures in England between 2005 and 2021. We observed individuals aged between 11 and 89 years for 17 survey years and grouped them into 17 five-year birth cohorts born between 1919 and 2008. Our descriptive results showed a steady increase in the prevalence of obesity over time for our four measures, but only WHtR and BMI showed very similar prevalence levels and trends during our study period. The prevalence of obesity increased with age using all obesity measures. This increase waslinear until the age of 80 when using central obesity measures. Conversely, BMI increased until around the age of 50 years, levelled off from 60 to 65 years, and then decreased afterwards. After the age of 80, all measures declined steeply, possibly due to low numbers at older ages and survivor bias. This pattern with age was confirmed in our APC analysis, in which the odds of obesity also increased with age and decreased among older adults when using BMI, though it continued to increase when using measures of central obesity. Period effects were similar across measures, with all showing increasing trends. However, high-risk BMI showed the smallest increase over time. Finally, cohort effects were similar across birth cohorts, with only the youngest birth cohorts showing lower odds of having obesity, but not always significantly different than 1.

Overall, period effects showed that after accounting for age and birth cohort, obesity increased only very slightly between 2005 and 2021 for the four measures of general and central obesity. This is in line with previous research, where BMI had a modest increase over time until around 2000 after which it levelled off [14]. Interestingly, we found very little evidence for cohort effects across the different measures, although there did appear to be a very slight trend to a reduction in the odds of obesity in for cohorts born more recently. We also found the odds of having high-risk WHtR was lower in women born after 2004, and for men born after 1974 when studying high-risk WHR. However, the odds were very similar between birth cohorts when studying high-risk BMI, which differs from an earlier study using data until from 1992 to 2019 [14]. This suggests that as cohorts have aged, they have become more similar, since both this study and the earlier study examined birth cohorts born between 1919 and 2008.

To our knowledge, this is the first study to compare age, period and cohort effects across multiple measures of general and central obesity. BMI was originally designed to measure obesity in a population and not to identify obesity in individuals [48]. WHtR and BMI show a very similar picture at the population level, as observed in our Fig. 1. However, they identify different groups of people as high-risk at an individual level, suggesting that both might be appropriate measures at a population level, but that they cannot both accurately identify risk in individuals, especially for the age groups at the tails of the age distribution.

This study supports the use of WHtR over BMI in the identification of obesity in individuals because we know that obesity-related health problems do not start to reduce in later life. This mirrors previous research that has found WHtR to be superior to other measures in adults [11, 49], older adults [50] and children [15]. Recently, alternative waist-to-height ratio (WHtR) data-driven thresholds have been proposed that differ from those used in our analysis. For example, Agbaje et al. suggest sex-specific pediatric thresholds [21], which were later reported as predictors of health outcomes in adults such as bone fracture and liver disease across diverse populations [15]. However, although we considered using these new thresholds in sensitivity analyses, for our sample, the Agbaje thresholds generated an unusually high proportion of older adults being classified as “high risk” (e.g. 85% of males over age 65 years) [15, 21]. The thresholds currently recommended by NICE suggest that high-risk is relative rare in English children and young adults (just over 6% in individuals aged 11–24 compared to around 17.5% using the paediatric thresholds), but we believe that from a public health perspective, this will help to identify children who are in the greatest need for treatment for obesity. Similarly, Cho et al. categorised BMI and central adiposity measures (including WC and WHtR) into tertiles, but the highest tertile corresponds to a much larger proportion of the population than would generally be considered as high risk [51]. This was especially concerning among children, where Cho et al. would classify 33% of children into high-risk [51]. The thresholds used in this study are extrapolated from adult data, and although this is a limitation, these are the current best practice guidelines [18, 44]. In the context of our research, they provide greater face validity than alternative thresholds developed using paediatric data extrapolated into adulthood or using percentiles. Future research could explore both BMI and central obesity measures using alternative data-driven thresholds, while carefully considering their face validity and comparability across age groups.

This study highlights that WHtR is a more universal measure of obesity and can provide consistent trends across men, women and children, as well as into older age. Given the superiority of WHtR over BMI, the implementation of WHtR as a standard measure for obesity should be encouraged both by clinicians when assessing overall health risk of obesity as well as in the wider community. The recently updated NICE guidelines on overweight and obesity management [18] should be disseminated more widely.

Our study used cross-sectional data, collected annually, allowing us to investigate population trends over a longer study period. However, there are some limitations to the data. First, the measures of central obesity were only continuously available since 2005, which restricted the years of comparability between general obesity and central obesity to the period 2005–2021. However, data for 2021 was available for analysis, allowing us to observe the youngest birth cohorts for a longer follow-up than previous research [14]. Second, age in years is only available for data prior to 2015, unless the user has successfully applied to the secure version of the data. We have imputed age data after this point, a method which has been outlined elsewhere [14, 38]. Although our main APC analysis did not include single years of age, the imputed age was used to determine the thresholds for obesity measures which are age- and sex-specific during childhood. This will inevitably have introduced some error, although we believe this to be minimal. Third, there was more missing data for central obesity measures, than for BMI measuring general obesity possibly due to the more invasive nature of waist measurements. In addition, in 2021 a smaller proportion of households participated in a nurse visit resulting in fewer observations with a valid waist circumference than in previous years [41]. Fourth, in 2021 only self-reported BMI was available, but the literature suggests the equation-adjusted version of self-reported BMI to be close to the physically measured BMI used in previous years [43]. Fifth, even though the sample is representative of England, the ethnic composition of the sample is 90% white and only 10% non-white, which does not allow analysis by ethnicity.

Our analysis used thresholds for each measure which were considered to indicate a high-risk of obesity-related diseases. However, it is important to keep in mind that obesity is a lifelong and relapsing disease and not simply a marker for other health problems. This has been highlighted in recent definitions of obesity published by the European Association for the Study of Obesity (EASO) [23] in 2024 and the Lancet Diabetes and Endocrinology Commission in 2025 [10]; both highlight how obesity is not merely a pre-curser to other diseases, but a distinct disease caused by excess adipose tissue. Further research is required to determine thresholds for BMI, WHtR and other obesity measures which relate directly to excess adiposity rather than the risks of comorbidities. Some research has been carried out in this area in children, where WHtR thresholds have been identified to predict DEXA measures of trunk fat and total fat mass [21]. The same WHtR thresholds have been shown to be associated with bone fracture and liver disease [15].

Results from this study can provide evidence towards stages in life where a subgroup analysis might be useful for targeted public health interventions. For example, our results suggest that obesity changes across different age groups but less so by sex. Further research should focus on exploring whether and how thresholds for these obesity measures differ across the lifecourse, particularly in relation to older adults, children and adolescents, and the clinical implications of these differences.

A significant strength of this study is the wide age range used to investigate obesity over the life course. Our analysis by age provided additional evidence to question the appropriateness of BMI when studying obesity in older adults. The reducing prevalence of high-risk BMI as people age contradicts what we know about obesity-related health risks, whereas other measures, particularly WHtR, showed a continuous linear increase in high-risk prevalence into older age. This suggests that central obesity might be a more appropriate indicator of risk across the life course supporting the updated NICE guidelines (NG246) [18] which recommend the use of WHtR alongside BMI. Our results also show that, between age, period and birth cohort, age plays a significant role in analysing long-term trends in obesity. While there is a slight but consistent increase in obesity over time, the similar odds across birth cohorts suggests that the substantial rise in obesity throughout an individual’s life could hinder the population’s capacity for healthy aging. Our findings support the use of early interventions, aimed at children and adolescents, to counteract the effects of aging in later life. They also support the use of WHtR, rather than BMI, to measure high risk, particularly in older adults.

## Supplementary information

Supplementary Material

## Data Availability

Data from the Health Survey for England (HSE) is freely available from the UK data service.

## Code availability

All analyses were carried out using Stata version 18 (StataCorp) [52]. Stata code used to generate the results from this study can be accessed from the authors at their discretion.
