# Supplementary material for: Long-term trends in central obesity in England: an age-period-cohort approach
Source: Int J Obes (Lond). 2025 Nov 17;50(3):519–26. doi: 10.1038/s41366-025-01949-5 (PMC12965872; doi:10.1038/s41366-025-01949-5)
Supplement: Supplementary file 1 — Supplementary Material [file 41366_2025_1949_MOESM1_ESM.docx]

**Supplementary Material**

**Sample Size**

Figure S1: Flowchart showing sample size for each measure of obesity

HSE 2005-2021 (n= 168,061)

Birth cohorts 1919-2008 (n = 120,024)

Valid BMI (n = 104,548)

Valid WC (n = 88,575)

Valid WHR (n = 88,331)

Valid WHtR (n = 84,033)

Age 11 to 89 (n = 144,046)

**Age-period-cohort Results**

Table S1: Age-period-cohort regression results: Estimated odds ratio (OR) of high-risk waist-to-height ratio (WHtR) compared to not high-risk and 95% confidence interval in females, Health Survey for England (2005-2021)

| High-risk WHtR | OR | 95% Confidence Interval | |  |  | OR | 95% Confidence Interval | |  |
| --- | --- | --- | --- | --- | --- | --- | --- | --- | --- |
| *HSE AGE GROUP* | | | |  | *3-YEAR PERIOD* | | | |  |
| 11-12 years | 0.4803 | 0.2845 | 0.8107 |  | 2005-2006 | 1 | - | - |  |
| 13-15 years | 0.3331 | 0.2049 | 0.5416 |  | 2007-2009 | 1.0830 | 1.0010 | 1.1717 |  |
| 16-17 years | 0.6302 | 0.4045 | 0.9818 |  | 2010-2012 | 1.2318 | 1.1135 | 1.3628 |  |
| 18-19 years | 1 | - | - |  | 2013-2015 | 1.2238 | 1.0829 | 1.3829 |  |
| 20-24 years | 1.3853 | 0.9936 | 1.9316 |  | 2016-2018 | 1.3837 | 1.1639 | 1.6451 |  |
| 25-29 years | 1.5086 | 1.0689 | 2.1233 |  | 2019-2021 | 1.4149 | 1.1631 | 1.7212 |  |
| 30-34 years | 1.8969 | 1.2990 | 2.7700 |  | *5-YEAR BIRTH COHORT* | | | |  |
| 35-39 years | 2.3170 | 1.5243 | 3.5219 |  | 1919-1923 | 1 | - | - |  |
| 40-44 years | 2.4835 | 1.5690 | 3.9311 |  | 1924-1928 | 0.9412 | 0.7022 | 1.2615 |  |
| 45-49 years | 3.2621 | 1.9715 | 5.3975 |  | 1929-1933 | 1.0163 | 0.7516 | 1.3743 |  |
| 50-54 years | 3.4606 | 1.9954 | 6.0016 |  | 1934-1938 | 1.0145 | 0.7218 | 1.4258 |  |
| 55-59 years | 3.7846 | 2.0801 | 6.8860 |  | 1939-1943 | 0.8735 | 0.5978 | 1.2765 |  |
| 60-64 years | 4.0783 | 2.1332 | 7.7972 |  | 1944-1948 | 0.7975 | 0.5235 | 1.2149 |  |
| 65-69 years | 4.7307 | 2.3527 | 9.5120 |  | 1949-1953 | 0.8200 | 0.5146 | 1.3064 |  |
| 70-74 years | 4.7990 | 2.2671 | 10.1586 |  | 1954-1958 | 0.7805 | 0.4661 | 1.3070 |  |
| 75-79 years | 5.2844 | 2.3641 | 11.8120 |  | 1959-1963 | 0.6957 | 0.3947 | 1.2263 |  |
| 80-84 years | 5.2754 | 2.2313 | 12.4724 |  | 1964-1968 | 0.7279 | 0.3922 | 1.3507 |  |
| 85-89 years | 4.9143 | 1.9500 | 12.3870 |  | 1969-1973 | 0.6894 | 0.3514 | 1.3523 |  |
|  |  |  |  |  | 1974-1978 | 0.6561 | 0.3159 | 1.3628 |  |
|  |  |  |  |  | 1979-1983 | 0.6036 | 0.2744 | 1.3277 |  |
|  |  |  |  |  | 1984-1988 | 0.6108 | 0.2618 | 1.4250 |  |
|  |  |  |  |  | 1989-1993 | 0.6173 | 0.2461 | 1.5485 |  |
|  |  |  |  |  | 1994-1998 | 0.4605 | 0.1696 | 1.2500 |  |
|  |  |  |  |  | 1999-2003 | 0.3532 | 0.1134 | 1.0994 |  |
|  |  |  |  |  | 2004-2008 | 0.1902 | 0.0499 | 0.7254 |  |

Table S2: Age-period-cohort regression results: Estimated odds (OR) of high-risk waist-to-height ratio (WHtR) compared to not high risk and 95% confidence interval in males, Health Survey for England (2005-2019)

| High-risk WHtR | OR | 95% Confidence Interval | |  |  | OR | 95% Confidence Interval | |  |
| --- | --- | --- | --- | --- | --- | --- | --- | --- | --- |
| *HSE AGE GROUP* | | | |  | *3-YEAR PERIOD* | | | |  |
| 11-12 years | 0.5722 | 0.3523 | 0.9292 |  | 2005-2006 | 1 | - | - |  |
| 13-15 years | 0.5625 | 0.3523 | 0.9292 |  | 2007-2009 | 1.0648 | .9777 | 1.1596 |  |
| 16-17 years | 0.5665 | 0.3744 | 0.8450 |  | 2010-2012 | 1.1379 | 1.0184 | 1.2714 |  |
| 18-19 years | 1 | - | - |  | 2013-2015 | 1.1917 | 1.0407 | 1.3647 |  |
| 20-24 years | 1.1306 | 0.7759 | 1.6475 |  | 2016-2018 | 1.1875 | .98337 | 1.4340 |  |
| 25-29 years | 1.4840 | 0.9958 | 2.2115 |  | 2019-2021 | 1.2496 | 1.0079 | 1.5494 |  |
| 30-34 years | 1.6910 | 1.0793 | 2.6494 |  | *5-YEAR BIRTH COHORT* | | | |  |
| 35-39 years | 2.4984 | 1.5372 | 4.0608 |  | 1919-1923 | 1 | - | - |  |
| 40-44 years | 2.8914 | 1.7062 | 4.8999 |  | 1924-1928 | 1.2633 | 0.9029 | 1.7677 |  |
| 45-49 years | 3.3298 | 1.8795 | 5.8992 |  | 1929-1933 | 1.3438 | 0.9520 | 1.8967 |  |
| 50-54 years | 3.8960 | 2.1002 | 7.2273 |  | 1934-1938 | 1.2851 | 0.8764 | 1.8843 |  |
| 55-59 years | 4.4541 | 2.2840 | 8.6860 |  | 1939-1943 | 1.3497 | 0.8840 | 2.0606 |  |
| 60-64 years | 4.6695 | 2.2757 | 9.5812 |  | 1944-1948 | 1.1750 | 0.7362 | 1.8753 |  |
| 65-69 years | 4.8519 | 2.2436 | 10.4924 |  | 1949-1953 | 1.1567 | 0.6909 | 1.9367 |  |
| 70-74 years | 5.3814 | 2.3578 | 12.2826 |  | 1954-1958 | 1.0505 | 0.5955 | 1.8534 |  |
| 75-79 years | 5.6003 | 2.3182 | 13.5294 |  | 1959-1963 | 0.9773 | 0.5245 | 1.8209 |  |
| 80-84 years | 5.7799 | 2.2523 | 14.8322 |  | 1964-1968 | 0.9139 | 0.4637 | 1.8013 |  |
| 85-89 years | 6.1549 | 2.2433 | 16.8872 |  | 1969-1973 | 0.7896 | 0.3781 | 1.6489 |  |
|  |  |  |  |  | 1974-1978 | 0.7177 | 0.3227 | 1.5959 |  |
|  |  |  |  |  | 1979-1983 | 0.6339 | 0.2672 | 1.5038 |  |
|  |  |  |  |  | 1984-1988 | 0.6397 | 0.2525 | 1.6206 |  |
|  |  |  |  |  | 1989-1993 | 0.5997 | 0.2165 | 1.6613 |  |
|  |  |  |  |  | 1994-1998 | 0.6933 | 0.2311 | 2.0798 |  |
|  |  |  |  |  | 1999-2003 | 0.7703 | 0.2349 | 2.5268 |  |
|  |  |  |  |  | 2004-2008 | .64207 | 0.1703 | 2.4205 |  |

Table S3: Age-period-cohort regression results: Estimated odds (OR) of high-risk waist-to hip ratio (WHR) compared to not high risk and 95% confidence interval in females, Health Survey for England (2005-2019)

| High-risk WHR | OR | 95% Confidence Interval | |  |  | OR | 95% Confidence Interval | |  |
| --- | --- | --- | --- | --- | --- | --- | --- | --- | --- |
| *HSE AGE GROUP* | | | |  | *3-YEAR PERIOD* | | | |  |
| 11-12 years | 1.2618 | 0.9082 | 1.7530 |  | 2005-2006 | 1 | - | - |  |
| 13-15 years | 0.9589 | 0.7152 | 1.2857 |  | 2007-2009 | 1.1362 | 1.0607 | 1.2171 |  |
| 16-17 years | 1.1739 | 0.8713 | 1.5817 |  | 2010-2012 | 1.3532 | 1.2383 | 1.4787 |  |
| 18-19 years | 1 | - | - |  | 2013-2015 | 1.4232 | 1.2771 | 1.5860 |  |
| 20-24 years | 1.0840 | 0.8300 | 1.4158 |  | 2016-2018 | 1.5559 | 1.3347 | 1.8138 |  |
| 25-29 years | 1.2803 | 0.9644 | 1.6996 |  | 2019-2021 | 1.5345 | 1.2878 | 1.8285 |  |
| 30-34 years | 1.7004 | 1.2425 | 2.3270 |  | *5-YEAR BIRTH COHORT* | | | |  |
| 35-39 years | 2.2137 | 1.5586 | 3.1442 |  | 1919-1923 | 1 | - | - |  |
| 40-44 years | 2.4567 | 1.6660 | 3.6229 |  | 1924-1928 | 1.0055 | 0.7808 | 1.2947 |  |
| 45-49 years | 3.1190 | 2.0302 | 4.7919 |  | 1929-1933 | 0.9872 | 0.7589 | 1.2843 |  |
| 50-54 years | 3.5855 | 2.2359 | 5.7495 |  | 1934-1938 | 1.0103 | 0.7482 | 1.3644 |  |
| 55-59 years | 3.8586 | 2.3047 | 6.4600 |  | 1939-1943 | 1.0108 | 0.7223 | 1.4144 |  |
| 60-64 years | 4.5105 | 2.5763 | 7.8968 |  | 1944-1948 | 0.9487 | 0.6531 | 1.3781 |  |
| 65-69 years | 4.7691 | 2.6014 | 8.7432 |  | 1949-1953 | 0.9851 | 0.6516 | 1.4894 |  |
| 70-74 years | 5.3679 | 2.7938 | 10.3137 |  | 1954-1958 | 0.9262 | 0.5861 | 1.4636 |  |
| 75-79 years | 6.1889 | 3.0637 | 12.5018 |  | 1959-1963 | 0.8584 | 0.5197 | 1.4179 |  |
| 80-84 years | 6.8465 | 3.2245 | 14.5373 |  | 1964-1968 | 0.7850 | 0.4540 | 1.3573 |  |
| 85-89 years | 7.1575 | 3.1867 | 16.0758 |  | 1969-1973 | 0.7384 | 0.4068 | 1.3402 |  |
|  |  |  |  |  | 1974-1978 | 0.6976 | 0.3653 | 1.3321 |  |
|  |  |  |  |  | 1979-1983 | 0.6964 | 0.3469 | 1.3977 |  |
|  |  |  |  |  | 1984-1988 | 0.6889 | 0.3256 | 1.4575 |  |
|  |  |  |  |  | 1989-1993 | 0.6498 | 0.2897 | 1.4574 |  |
|  |  |  |  |  | 1994-1998 | 0.4911 | 0.2059 | 1.1717 |  |
|  |  |  |  |  | 1999-2003 | 0.4472 | 0.1736 | 1.1519 |  |
|  |  |  |  |  | 2004-2008 | 0.3514 | 0.1244 | 0.9923 |  |

Table S4: Age-period-cohort regression results: Estimated odds (OR) of high-risk waist-to hip ratio (WHR)compared to not high risk and 95% confidence interval in males, Health Survey for England (2005-2019)

| High-risk WHR | OR | 95% Confidence Interval | |  |  | OR | 95% Confidence Interval | |  |
| --- | --- | --- | --- | --- | --- | --- | --- | --- | --- |
| *HSE AGE GROUP* | | | |  | *3-YEAR PERIOD* | | | |  |
| 11-12 years | 1.0103 | 0.7268 | 1.4044 |  | 2005-2006 | 1 | - | - |  |
| 13-15 years | 0.9296 | 0.7029 | 1.2293 |  | 2007-2009 | 1.0932 | 1.0037 | 1.1907 |  |
| 16-17 years | 0.6981 | 0.5116 | 0.9525 |  | 2010-2012 | 1.2613 | 1.1266 | 1.4122 |  |
| 18-19 years | 1 | - | - |  | 2013-2015 | 1.2703 | 1.1062 | 1.4587 |  |
| 20-24 years | 1.2155 | 0.9339 | 1.5821 |  | 2016-2018 | 1.4510 | 1.1938 | 1.7635 |  |
| 25-29 years | 1.8728 | 1.4046 | 2.4970 |  | 2019-2021 | 1.6178 | 1.2889 | 2.0308 |  |
| 30-34 years | 2.8516 | 2.0441 | 3.9781 |  | *5-YEAR BIRTH COHORT* | | | |  |
| 35-39 years | 3.8991 | 2.6642 | 5.7063 |  | 1919-1923 | 1 | - | - |  |
| 40-44 years | 4.5207 | 2.9329 | 6.9680 |  | 1924-1928 | 1.4986 | 1.0188 | 2.2042 |  |
| 45-49 years | 4.8744 | 2.9833 | 7.9643 |  | 1929-1933 | 1.2216 | 0.8258 | 1.8070 |  |
| 50-54 years | 5.6544 | 3.2673 | 9.7857 |  | 1934-1938 | 1.4037 | 0.9066 | 2.1734 |  |
| 55-59 years | 6.4491 | 3.5080 | 11.8558 |  | 1939-1943 | 1.4716 | 0.9079 | 2.3854 |  |
| 60-64 years | 6.6312 | 3.3940 | 12.9561 |  | 1944-1948 | 1.1895 | 0.7007 | 2.0192 |  |
| 65-69 years | 6.9974 | 3.3497 | 14.6171 |  | 1949-1953 | 1.1877 | 0.6655 | 2.1196 |  |
| 70-74 years | 6.4112 | 2.8845 | 14.2498 |  | 1954-1958 | 0.9467 | 0.5044 | 1.7769 |  |
| 75-79 years | 6.8663 | 2.8782 | 16.3802 |  | 1959-1963 | 0.8136 | 0.4114 | 1.6090 |  |
| 80-84 years | 6.2419 | 2.4479 | 15.9163 |  | 1964-1968 | 0.6863 | 0.3289 | 1.4320 |  |
| 85-89 years | 5.3255 | 1.9293 | 14.7001 |  | 1969-1973 | 0.5223 | 0.2364 | 1.1542 |  |
|  |  |  |  |  | 1974-1978 | 0.3831 | 0.1637 | 0.8966 |  |
|  |  |  |  |  | 1979-1983 | 0.3195 | 0.1285 | 0.7943 |  |
|  |  |  |  |  | 1984-1988 | 0.2926 | 0.1107 | 0.7734 |  |
|  |  |  |  |  | 1989-1993 | 0.2365 | 0.0835 | 0.6698 |  |
|  |  |  |  |  | 1994-1998 | 0.2171 | 0.0716 | 0.6590 |  |
|  |  |  |  |  | 1999-2003 | 0.2078 | 0.0636 | 0.6792 |  |
|  |  |  |  |  | 2004-2008 | 0.1440 | 0.0400 | 0.5184 |  |

Table S5: Age-period-cohort regression results: Estimated odds (OR) of high-risk waist circumference (WC) compared to not high risk and 95% confidence interval in females, Health Survey for England (2005-2019)

| High-risk WC | OR | 95% Confidence Interval | |  |  | OR | 95% Confidence Interval | |  |
| --- | --- | --- | --- | --- | --- | --- | --- | --- | --- |
| *HSE AGE GROUP* | | | |  | *3-YEAR PERIOD* | | | |  |
| 11-12 years | 1.1596 | 0.8642 | 1.5559 |  | 2005-2006 | 1 | - | - |  |
| 13-15 years | 0.9844 | 0.7648 | 1.2671 |  | 2007-2009 | 1.0883 | 1.0194 | 1.1618 |  |
| 16-17 years | 1.2217 | 0.9389 | 1.5897 |  | 2010-2012 | 1.2050 | 1.1066 | 1.3122 |  |
| 18-19 years | 1 | - | - |  | 2013-2015 | 1.1407 | 1.0276 | 1.2663 |  |
| 20-24 years | 1.4812 | 1.1749 | 1.8673 |  | 2016-2018 | 1.2365 | 1.0662 | 1.4341 |  |
| 25-29 years | 1.8140 | 1.4141 | 2.3272 |  | 2019-2021 | 1.2625 | 1.0670 | 1.4938 |  |
| 30-34 years | 2.4735 | 1.8674 | 3.2762 |  | *5-YEAR BIRTH COHORT* | | | |  |
| 35-39 years | 2.9039 | 2.1150 | 3.9869 |  | 1919-1923 | 1 | - | - |  |
| 40-44 years | 3.4255 | 2.4032 | 4.8826 |  | 1924-1928 | 1.2613 | 0.9846 | 1.6157 |  |
| 45-49 years | 4.1429 | 2.7896 | 6.1527 |  | 1929-1933 | 1.4252 | 1.0997 | 1.8470 |  |
| 50-54 years | 4.8173 | 3.1081 | 7.4665 |  | 1934-1938 | 1.4318 | 1.0647 | 1.9254 |  |
| 55-59 years | 5.1586 | 3.1897 | 8.3430 |  | 1939-1943 | 1.3299 | 0.9549 | 1.8523 |  |
| 60-64 years | 6.1616 | 3.6437 | 10.4196 |  | 1944-1948 | 1.3159 | 0.9112 | 1.9003 |  |
| 65-69 years | 6.9046 | 3.9004 | 12.2229 |  | 1949-1953 | 1.4048 | 0.9361 | 2.1081 |  |
| 70-74 years | 7.3087 | 3.9410 | 13.5542 |  | 1954-1958 | 1.3233 | 0.8454 | 2.0714 |  |
| 75-79 years | 7.3072 | 3.7483 | 14.2451 |  | 1959-1963 | 1.2280 | 0.7521 | 2.0050 |  |
| 80-84 years | 7.4765 | 3.6502 | 15.3139 |  | 1964-1968 | 1.2199 | 0.7156 | 2.0799 |  |
| 85-89 years | 6.6602 | 3.0755 | 14.4228 |  | 1969-1973 | 1.3028 | 0.7300 | 2.3251 |  |
|  |  |  |  |  | 1974-1978 | 1.2403 | 0.6627 | 2.3210 |  |
|  |  |  |  |  | 1979-1983 | 1.2543 | 0.6395 | 2.4602 |  |
|  |  |  |  |  | 1984-1988 | 1.3187 | 0.6399 | 2.7177 |  |
|  |  |  |  |  | 1989-1993 | 1.3923 | 0.6398 | 3.0300 |  |
|  |  |  |  |  | 1994-1998 | 1.0903 | 0.4743 | 2.5063 |  |
|  |  |  |  |  | 1999-2003 | 1.1120 | 0.4529 | 2.7301 |  |
|  |  |  |  |  | 2004-2008 | 0.8830 | 0.3125 | 2.4946 |  |

Table S6: Age-period-cohort regression results: Estimated odds (OR) of high-risk waist circumference (WC) compared to not high risk and 95% confidence interval in males, Health Survey for England (2005-2019)

| High-risk WC | OR | 95% Confidence Interval | |  |  | OR | 95% Confidence Interval | |  |
| --- | --- | --- | --- | --- | --- | --- | --- | --- | --- |
| *HSE AGE GROUP* | | | |  | *3-YEAR PERIOD* | | | |  |
| 11-12 years | 0.9064 | 0.6246 | 1.3154 |  | 2005-2006 | 1 | - | - |  |
| 13-15 years | 0.9655 | 0.7015 | 1.3290 |  | 2007-2009 | 1.1053 | 1.0238 | 1.1932 |  |
| 16-17 years | 0.8654 | 0.6124 | 1.2229 |  | 2010-2012 | 1.2116 | 1.0969 | 1.3381 |  |
| 18-19 years | 1 | - | - |  | 2013-2015 | 1.2483 | 1.1052 | 1.4100 |  |
| 20-24 years | 1.1721 | 0.8593 | 1.5987 |  | 2016-2018 | 1.3246 | 1.1171 | 1.5706 |  |
| 25-29 years | 1.6775 | 1.2111 | 2.3236 |  | 2019-2021 | 1.4932 | 1.2270 | 1.8171 |  |
| 30-34 years | 2.0885 | 1.4492 | 3.0098 |  | *5-YEAR BIRTH COHORT* | | | |  |
| 35-39 years | 2.5301 | 1.6932 | 3.7807 |  | 1919-1923 | 1 | - | - |  |
| 40-44 years | 2.6064 | 1.6766 | 4.0520 |  | 1924-1928 | 1.5729 | 1.1668 | 2.1203 |  |
| 45-49 years | 2.8565 | 1.7612 | 4.6331 |  | 1929-1933 | 1.5390 | 1.1289 | 2.0981 |  |
| 50-54 years | 3.1107 | 1.8351 | 5.2728 |  | 1934-1938 | 1.4733 | 1.0425 | 2.0820 |  |
| 55-59 years | 3.1927 | 1.7975 | 5.6709 |  | 1939-1943 | 1.4428 | 0.9838 | 2.1160 |  |
| 60-64 years | 3.0978 | 1.6625 | 5.7723 |  | 1944-1948 | 1.2270 | 0.8035 | 1.8736 |  |
| 65-69 years | 2.8634 | 1.4626 | 5.6058 |  | 1949-1953 | 1.2057 | 0.7558 | 1.9232 |  |
| 70-74 years | 2.8561 | 1.3872 | 5.8804 |  | 1954-1958 | 0.9598 | 0.5740 | 1.6051 |  |
| 75-79 years | 2.4905 | 1.1470 | 5.4074 |  | 1959-1963 | 0.8487 | 0.4833 | 1.4902 |  |
| 80-84 years | 2.3004 | 1.0015 | 5.2838 |  | 1964-1968 | 0.8029 | 0.4349 | 1.4826 |  |
| 85-89 years | 2.2578 | 0.9245 | 5.5141 |  | 1969-1973 | 0.6652 | 0.3417 | 1.2949 |  |
|  |  |  |  |  | 1974-1978 | 0.5339 | 0.2600 | 1.0963 |  |
|  |  |  |  |  | 1979-1983 | 0.4739 | 0.2178 | 1.0309 |  |
|  |  |  |  |  | 1984-1988 | 0.4565 | 0.1982 | 1.0515 |  |
|  |  |  |  |  | 1989-1993 | 0.4468 | 0.1810 | 1.1034 |  |
|  |  |  |  |  | 1994-1998 | 0.4333 | 0.1639 | 1.1454 |  |
|  |  |  |  |  | 1999-2003 | 0.4664 | 0.1649 | 1.3197 |  |
|  |  |  |  |  | 2004-2008 | 0.4065 | 0.1311 | 1.2608 |  |

Table S7: Age-period-cohort regression results: Estimated odds (OR) of high-risk body mass index (BMI) compared to not high risk and 95% confidence interval in females, Health Survey for England (2005-2019)

| High-risk BMI | OR | 95% Confidence Interval | |  |  | OR | 95% Confidence Interval | |  |
| --- | --- | --- | --- | --- | --- | --- | --- | --- | --- |
| *HSE AGE GROUP* | | | |  | *3-YEAR PERIOD* | | | |  |
| 11-12 years | 0.8307 | 0.5915 | 1.1666 |  | 2005-2006 | 1 | - | - |  |
| 13-15 years | 0.6703 | 0.5038 | 0.8918 |  | 2007-2009 | 1.0244 | 0.9579 | 1.0955 |  |
| 16-17 years | 0.9036 | 0.6747 | 1.2103 |  | 2010-2012 | 1.0991 | 1.0082 | 1.1983 |  |
| 18-19 years | 1 | - | - |  | 2013-2015 | 1.0948 | 0.9847 | 1.2172 |  |
| 20-24 years | 1.6740 | 1.3240 | 2.1165 |  | 2016-2018 | 1.2131 | 1.0429 | 1.4111 |  |
| 25-29 years | 1.9705 | 1.5281 | 2.5409 |  | 2019-2021 | 1.1935 | 1.0035 | 1.4194 |  |
| 30-34 years | 2.5292 | 1.8997 | 3.3673 |  | *5-YEAR BIRTH COHORT* | | | |  |
| 35-39 years | 2.9579 | 2.1417 | 4.0852 |  | 1919-1923 | 1 | - | - |  |
| 40-44 years | 3.3037 | 2.3047 | 4.7359 |  | 1924-1928 | 1.1050 | 0.8075 | 1.5122 |  |
| 45-49 years | 3.8494 | 2.5784 | 5.7469 |  | 1929-1933 | 1.2099 | 0.8782 | 1.6670 |  |
| 50-54 years | 3.9260 | 2.5190 | 6.1189 |  | 1934-1938 | 1.2007 | 0.8449 | 1.7065 |  |
| 55-59 years | 3.9636 | 2.4355 | 6.4505 |  | 1939-1943 | 1.0666 | 0.7289 | 1.5607 |  |
| 60-64 years | 3.8619 | 2.2687 | 6.5739 |  | 1944-1948 | 1.0007 | 0.6626 | 1.5115 |  |
| 65-69 years | 3.9978 | 2.2451 | 7.1191 |  | 1949-1953 | 0.9574 | 0.6121 | 1.4974 |  |
| 70-74 years | 3.6030 | 1.9316 | 6.7208 |  | 1954-1958 | 0.9084 | 0.5586 | 1.4772 |  |
| 75-79 years | 3.4521 | 1.7622 | 6.7627 |  | 1959-1963 | 0.8555 | 0.5053 | 1.4484 |  |
| 80-84 years | 2.3511 | 1.1392 | 4.8522 |  | 1964-1968 | 0.8600 | 0.4875 | 1.5169 |  |
| 85-89 years | 1.9045 | 0.8624 | 4.2056 |  | 1969-1973 | 0.9281 | 0.5037 | 1.7100 |  |
|  |  |  |  |  | 1974-1978 | 0.8817 | 0.4572 | 1.7004 |  |
|  |  |  |  |  | 1979-1983 | 0.9420 | 0.4672 | 1.8993 |  |
|  |  |  |  |  | 1984-1988 | 1.0030 | 0.4734 | 2.1249 |  |
|  |  |  |  |  | 1989-1993 | 1.0137 | 0.4536 | 2.2655 |  |
|  |  |  |  |  | 1994-1998 | 0.9016 | 0.3815 | 2.1309 |  |
|  |  |  |  |  | 1999-2003 | 0.8205 | 0.3207 | 2.0993 |  |
|  |  |  |  |  | 2004-2008 | 1.1050 | 0.8075 | 1.5122 |  |

Table S8: Age-period-cohort regression results: Estimated odds (OR) of high-risk body mass index (BMI) compared to not high risk and 95% confidence interval in males, Health Survey for England (2005-2019)

| High-risk BMI | OR | 95% Confidence Interval | |  |  | OR | 95% Confidence Interval | |  |
| --- | --- | --- | --- | --- | --- | --- | --- | --- | --- |
| *HSE AGE GROUP* | | | |  | *3-YEAR PERIOD* | | | |  |
| 11-12 years | 0.5471 | 0.3794 | 0.7888 |  | 2005-2006 | 1 | - | - |  |
| 13-15 years | 0.6319 | 0.4698 | 0.8500 |  | 2007-2009 | 1.0657 | 0.9890 | 1.1484 |  |
| 16-17 years | 0.6980 | 0.5133 | 0.9493 |  | 2010-2012 | 1.1758 | 1.0676 | 1.2950 |  |
| 18-19 years | 1 | - | - |  | 2013-2015 | 1.2676 | 1.1255 | 1.4277 |  |
| 20-24 years | 1.1152 | 0.8587 | 1.4484 |  | 2016-2018 | 1.3544 | 1.1448 | 1.6025 |  |
| 25-29 years | 1.6116 | 1.2093 | 2.1477 |  | 2019-2021 | 1.3870 | 1.1415 | 1.6853 |  |
| 30-34 years | 1.6765 | 1.2060 | 2.3304 |  | *5-YEAR BIRTH COHORT* | | | |  |
| 35-39 years | 2.0462 | 1.4181 | 2.9525 |  | 1919-1923 | 1 | - | - |  |
| 40-44 years | 1.9104 | 1.2699 | 2.8739 |  | 1924-1928 | 1.2863 | 0.8752 | 1.8904 |  |
| 45-49 years | 2.1805 | 1.3886 | 3.4240 |  | 1929-1933 | 1.3736 | 0.9308 | 2.0270 |  |
| 50-54 years | 2.1703 | 1.3218 | 3.5633 |  | 1934-1938 | 1.3768 | 0.9061 | 2.0919 |  |
| 55-59 years | 2.1203 | 1.2312 | 3.6515 |  | 1939-1943 | 1.3346 | 0.8519 | 2.0907 |  |
| 60-64 years | 1.8836 | 1.0427 | 3.4028 |  | 1944-1948 | 1.1718 | 0.7237 | 1.8975 |  |
| 65-69 years | 1.5931 | 0.8392 | 3.0240 |  | 1949-1953 | 1.1519 | 0.6857 | 1.9350 |  |
| 70-74 years | 1.4783 | 0.7406 | 2.9509 |  | 1954-1958 | 1.0351 | 0.5918 | 1.8103 |  |
| 75-79 years | 1.1633 | 0.5528 | 2.4482 |  | 1959-1963 | 0.9632 | 0.5276 | 1.7584 |  |
| 80-84 years | 0.9848 | 0.4410 | 2.1990 |  | 1964-1968 | 0.8996 | 0.4711 | 1.7179 |  |
| 85-89 years | 0.8569 | 0.3581 | 2.0505 |  | 1969-1973 | 0.8153 | 0.4081 | 1.6287 |  |
|  |  |  |  |  | 1974-1978 | 0.6595 | 0.3143 | 1.3836 |  |
|  |  |  |  |  | 1979-1983 | 0.5931 | 0.2688 | 1.3088 |  |
|  |  |  |  |  | 1984-1988 | 0.5387 | 0.2314 | 1.2544 |  |
|  |  |  |  |  | 1989-1993 | 0.5245 | 0.2111 | 1.3030 |  |
|  |  |  |  |  | 1994-1998 | 0.5567 | 0.2102 | 1.4747 |  |
|  |  |  |  |  | 1999-2003 | 0.5818 | 0.2039 | 1.6600 |  |
|  |  |  |  |  | 2004-2008 | 0.5359 | 0.1690 | 1.6993 |  |

**Sensitivity Analysis**

Figure S2: Estimated odds ratio (OR) and 95% confidence intervals on displayed a logarithmic scale for high-risk in 1) waist-to-height ratio (WHtR), 2) waist-to-hip ratio (WHR), 3) waist circumference (WC) and 4) high-risk body mass index (BMI) by HSE age group, four-year survey period, and ten-year birth cohort for males and females (Health Survey England, 2005-2021)

| *1. High-risk waist-to-height ratio (WHtR)* | | |
| --- | --- | --- |
| a. Age | b. Period | c. Birth cohort |
|  |  |  |
| *2. High-risk waist-to-hip ratio (WHR)* | | |
| a. Age | b. Period | c. Birth cohort |
|  |  |  |
| *3. High-risk waist circumference (WC)* | | |
| a. Age | b. Period | c. Birth cohort |
|  |  |  |
| *4. High-risk body mass index (BMI)* | | |
| a. Age | b. Period | c. Birth cohort |
|  |  |  |
